# The Influence of the Addition of Nuts on the Thermal and Rheological Properties of Wheat Flour

**DOI:** 10.3390/molecules26133969

**Published:** 2021-06-29

**Authors:** Karolina Pycia, Lesław Juszczak

**Affiliations:** 1 Department of Food Technology and Human Nutrition, Institute of Food Technology and Nutrition, College of Natural Sciences, University of Rzeszow, Zelwerowicza 4 St., 35-601 Rzeszow, Poland; 2 Department of Food Analysis and Evaluation of Food Quality, University of Agriculture in Krakow, Balicka 122, 30-149 Krakow, Poland; rrjuszcz@cyf-kr.edu.pl

**Keywords:** flour, nuts, thermodynamic properties, rheological properties, differential scanning calorimetry

## Abstract

The aim of the study was to assess the influence of replacing wheat flour with hazelnuts or walnuts, in various amounts, on the thermal and rheological properties of the obtained systems. The research material were systems in which wheat flour was replaced with ground hazelnuts (H) or walnuts (W) in the amount of 5%, 10%, and 15%. The parameters of the thermodynamic gelatinization characteristics were determined by the differential scanning calorimetry method. In addition, the pasting characteristics were determined with the use of a viscosity analyzer and the viscoelastic properties were assessed. Sweep frequency and creep and recovery tests were used to assess the viscoelastic properties of the tested gels. It was found that replacing wheat flour with nuts increased the values of gelatinization temperature, gelatinization, and retrogradation enthalpy, and the degree of retrogradation. The highest viscosity was characteristic of the control sample (2039 mPa·s), and the lowest for the paste with 15% addition of walnuts (1120 mPa·s). Replacing the flour with nuts resulted in a very visible reduction in the viscosity of such systems. In addition, gels based on the systems with the addition of H and W were weak gels (tan δ = *G*″/*G*′ > 0.1), and the values of *G*′ and *G*″ parameters decreased with the increased share of nuts in the systems. Creep and recovery analysis indicated that the systems in which wheat flour was replaced with hazelnuts were less susceptible to deformation compared to the systems with the addition of W.

## 1. Introduction

Wheat grain is one of the basic bread grains in the world. Globally, over 60% of wheat production is used for consumption [1]. Apart from maize, it is also the basic raw material in the milling industry, in the baking and confectionery industry to the production of pasta and for obtaining animal feed. The composition and properties of wheat flour determine the course of technological processes, as well as the quality, nutritional value, and health-promoting values of the obtained products. Technological usefulness of flour is a function of factors, such as the variety and origin of grain, growing conditions, structure, and amount of fertilization, grain quality, storage conditions, method of grain grinding, and baking value of flour. The flour baking ability is a set of flour features that ensures the appropriate fermentation capacity of the dough based on it, retention of fermentation gases and obtaining the appropriate shape of bread, as well as the appropriate texture of its crumb [2]. The baking value of flour is checked by direct and indirect methods. Carrying out a trial baking and evaluation of the received bread is part of the direct method. Indirect methods, on the other hand, involve the assessment of the chemical composition of flour and its physical properties [3]. In the analysis of physical characteristics, the rheological properties of the flour and the dough obtained, which result from the chemical composition of the flour, are of the greatest importance. Knowledge of the rheological properties of flour and dough allows for proper planning of bread production and affects its final quality [4]. The chemical composition of flour is dominated by starch. There are also non-starch ingredients, such as protein, fat, and minerals. However, the presence of other non-starch ingredients significantly influences the thermal and rheological properties of starch. However, wheat flour used to make bread has a reduced nutritional value due to the grinding process in which it is made. Highly extracted flour is devoid of bran and other valuable grain ingredients. Therefore, it is characterized by a low content of dietary fiber, bioactive ingredients, and minerals [5,6]. Therefore, in order to improve the nutritional value of bread and give it health benefits, part of the flour can be replaced with a raw material with a high health-promoting potential, which may be fruit, vegetables, as well as nuts or products based on them. According to Pycia and Ivanišová [7], Pycia and Kapusta [8], enriching wheat bread with walnuts and hazelnuts, with different degrees of maturity, has a positive effect on its nutritional value and antioxidant properties, resulting in a reduction in the volume of the loaves. This is due to the chemical composition of nuts and the related health-promoting properties. Walnuts are a rich source of polyunsaturated fatty acids; they show the most favorable ratio of n-6 and n-3 fatty acids, which should be 4:1. Hazelnuts and almonds, on the other hand, are an excellent source of vitamin E [9]. As reported by Kornsteiner et al. [10], a portion of hazelnuts (42 g) provides over 100% of the recommended daily intake of vitamin E, thanks to which the body is protected against free radicals, thus preventing the aging process, the development of atherosclerosis and cancer. Replacing a part of the wheat flour with nuts will probably affect the starch pasting method and the rheological properties of the paste based on this flour and, thus, the rheology of the dough and the quality of the bread obtained. Nevertheless, the literature on the subject lacks information on the type and direction of thermal and rheological properties of system based of wheat flour and nuts.

The aim of the study was to assess the effect of replacing wheat flour with hazelnuts and walnuts on the characteristics of starch gelatinization, as well as rheological and thermal properties of these systems.

## 2. Materials and Methods

### 2.1. Research Material

The research material were mixtures (systems) of type 650 universal wheat flour (Gdańskie Młyny, Poland) with the addition of ground hazelnuts (H) and walnuts (W) from the 2020 harvest. The nuts were purchased on the local market in Rzeszow (Podkarpackie, Poland). In the developed mixtures, wheat flour (WF) was replaced with hazelnuts and walnuts in the amount of 5% (WFH5%, WFW5%), 10% (WFH10%, WFW10%), and 15% (WFH15%, WFW15%). The control sample was wheat flour without the addition of nuts (control). In the examined systems, the starch content was determined by the Ewers method [11], which was respectively 72.9 g/100 g d.m. (control), 70.5 g/100 g d.m. (WFH5%), 65.8 g/100 g d.m. (WFH10%), 60.9 g/100 g d.m. (WFH15%), 70.7 g/100 g d.m. (WFW5%), 66.5 g/100 g d.m. (WFW10%), 60.7 g/100 g d.m. (WFW15%). The dry matter content in the prepared mixtures was determined using the AACC method [12].

### 2.2. Methods

#### 2.2.1. Thermodynamic Characteristics of Gelatinization

Thermodynamic gelatinization characteristics were determined with the use of F204 Phoenix Differential Scanning Calorimeter (DSC) (Netzsch, Germany). The mixture of the analyzed flour/nuts and water systems (1:3) was hermetically sealed in aluminum containers and left for 24 h to moisturize in room temperature (about 23 °C). Then the samples were heated in a calorimeter in the temperature range 25–100 °C at a rate of 10 °C/min. An empty calorimeter cup was used as a standard. From the gelatinization thermograms, the onset (T_O_), the peak (T_P_) and the end (T_E_) transition temperatures and the gelatinization enthalpy ΔH_G_ (J/g) were determined. The samples after gelatinization were stored for 7 days at 5 ± 1 °C and scanned in the calorimeter in an analogous manner. The following temperatures were determined from the thermograms: the beginning T_O_, the T_P_ peak, the end of the T_E_ peak, and the retrogradation enthalpy ΔH_R_ (J/g). The R coefficient, i.e., the percentage of retrogradation (ΔH_R_/ΔH_G_) × 100, was also calculated. The determination was made in triplicate.

#### 2.2.2. Characteristics of Pasting of the Analyzed Systems

The pasting characteristics of 5% suspensions of the tested systems were performed using the RVA viscosity analyzer (Rapid Visco Analyzer, Tec Master, Perten Instruments, Sweden). Samples (all the time mixed at 160 rpm/min) were kept at 50 °C for 1 min, then heated to 95 °C at a rate of 12 °C/min, further kept at 95 °C for 5 min, cooled down to temperature of 50 °C at a rate of 12 °C/min and finally kept at 50 °C for 2 min. The following parameters were read from the obtained viscograms: pasting temperature (PT) (°C), maximum viscosity during heating (PV) (mPa·s), viscosity at 95 °C (HPV) (mPa·s), final viscosity at 50 °C (FV) (mPa·s), decrease in viscosity during heating, value: PV-HPV (BD) (mPa·s), increase in viscosity during cooling, value: FV-HPV (SB) (mPa·s). The determination was made in triplicate.

#### 2.2.3. Frequency Sweep Test

The viscoelastic properties of the analyzed systems were characterized at a temperature of 25 °C using a MARS II oscillating rheometer (Thermo Fisher Scientific, Waltham, MA, USA) equipped with a system of parallel plates (diameter 35 mm, gap size 1 mm). The paste samples obtained in the RVA analyzer (Section 2.2.1) were placed in the rheometer measuring system and left for 2 min to relax stresses and stabilize temperature.

The sweep frequency curves were determined in the range of linear viscoelasticity at a constant strain amplitude of 0.1% in the angular velocity range 1–100 rad/s. The experimental data were described by the power equations:

(1) $$G^{\prime }\left(\omega \right)=K^{\prime }\cdot \omega^{n^{\prime }}$$

(2) $$G^{\prime \prime }\left(\omega \right)=K^{\prime \prime }\cdot \omega^{n^{\prime \prime }}$$

where: *G*′—storage modulus (Pa), *G*″—loss modulus (Pa), *ω*—angular frequency (rad/s), *K*′ *K*″, *n*′, *n*″—experimental constants.

The determination was made in triplicate.

#### 2.2.4. Creep and Recovery Test

Creep and recovery tests were performed with constant creep deformation τ_0_ = 2 Pa for 120 s. The recovery phase lasted 240 s. Experimental data were described using the Burgers model:

(3) $$J\left(t\right)=J_{0}+\frac{t}{\eta_{0}}+J_{1}\cdot \left(1-\exp^{-t/\lambda_{ret}}\right)for the creep phase$$

(4) $$J(t)=\frac{t_{1}}{\eta_{0}}-J_{1}\cdot (1-{exp}^{t_{1}/\lambda_{ret}})\cdot {exp}^{-t/\lambda_{ret}}\;\mathrm{for}\;\mathrm{the}\;\mathrm{recovery}\;\mathrm{phase}$$

where: *J*—susceptibility (Pa^−1^), *J*_0_—immediate susceptibility (Pa^−1^), *J*_1_—viscoelastic compliance (Pa^−1^), *η*_0_—zero shear viscosity (Pa∙s), *λ_ret_*—retardation time (s), *t*_1_—time after which the stress is removed (s).

The determination was made in duplicate.

#### 2.2.5. Statistical Analysis

The obtained results were subjected to a statistical analysis including a two-way ANOVA. In order to determine the significance of differences between the mean values, Duncan’s test was performed at the significance level *p* = 0.05. In addition, between the parameters characterizing the properties of nuts, the values of Pearson’s linear correlation coefficients were calculated, the significance of which was tested at the significance level *p* = 0.05. Statistical analysis was performed using the Statistica 13.3 program (StatSoft, Tulsa, Poland).

## 3. Results and Discussion

### 3.1. Gelatinization Properties

Table 1 summarizes the parameters of the thermal gelatinization and retrogradation characteristics determined by the differential scanning calorimetry (DSC) method. DSC enables the determination of gelatinization characteristics of starch systems in the presence of other components, such as protein or lipids. This technique allows for the interpretation of the interaction between all the ingredients of the dough. The performed two-way analysis of variance confirmed only a statistically significant influence of the type of nuts added to the flour on the value of the initial temperature and the temperature of the gelatinization peak. It was found that the control sample was characterized by the highest value of the transformation initiation temperature T_O_, and the presence of nuts in the system resulted in an increase in the value of this parameter (Table 1). The mean T_O_ value of the systems containing hazelnuts (WFH) and walnuts (WFW) was higher by 0.9 °C and 1.8 °C, respectively, compared to the control sample. In turn, the mean value of T_P_ of the WFH and WFW systems was higher than the value of this parameter for the control sample by 0.4 °C and 0.9 °C, respectively. The end-of-conversion temperature of T_E_ ranged from 69.7 °C (control) to 71.3 °C (WFW15%). The presence of nuts in the analyzed systems increased the value of T_E_. Agyare et al. [13] also found an increase in gelatinization temperatures in wheat dough to which various structured fats were added. Increasing gelatinization temperatures of flour systems with the addition of nuts may result from their different ratio to water in the present fat.

The fat present in the system hinders the swelling of starch grains, thereby increasing the gelatinization temperature and delays the starch gelatinization process. Increasing the degree of starch crystallinity increases the gelatinization temperature. The gelatinization characteristics of starch depend on the size of the starch grains. Because Chiotella and Le Meste [14] indicate that small, starch grains gel at a higher temperature and are characterized by a wider gelatinization temperature range than large grain starches, which may be due to the weaker ordering of polysaccharide chains in smaller grains compared to larger ones. The gelatinization enthalpy ΔH_G_ depended statistically significantly on the type and level of added nuts (*p* < 0.001). The lowest value of gelatinization enthalpy was characteristic for the control sample, and the highest value for the WFH15% system. Replacing flour with nuts increased the value of this parameter. The enthalpy of gelatinization is a function of many factors, such as the shape and size of the grains and the phosphorus content. This parameter is a measure of the amount of energy necessary to disintegrate the ordered structure of the starch grain and depends, inter alia, on the availability of water, i.e., the starch/water ratio [15]. Therefore, increasing the value of this parameter in systems with the addition of nuts rich in fat probably makes it difficult for the starch to absorb water. Therefore, it makes its pasting difficult. Yasui et al. [16] noted that amylopectin is the dominant factor influencing the gelatinization enthalpy value due to its degree of polymerization. When the paste is stored, unfavorable changes occur in it related to the retrogradation process. Retrogradation is a phenomenon that results from the formation of hydrogen bonds between the amylose and amylopectin chains and the association of starch polymers. During this process, amylose forms double helices of 40–70 glucose molecules [15,17]. By subjecting the starch gel, which has been stored under refrigeration to the heating process, an endothermic peak is observed on the thermogram, which is a measure of the amount of energy necessary for the disintegration of recrystallized amylopectin [15]. According to Singh et al. [17] and Pycia et al. [15] this endothermic peak generally has a lower range of about 10–20 °C in comparison with gelatinization temperatures. Table 1 presents parameters illustrating the thermal changes of the starch gel observed after the retrogradation process. The studies did not show a statistically significant influence of the type of added nuts, the level of supplementation and the interaction between these factors on the value of T_O_ and T_P_ in the analyzed systems. The control sample was characterized by the highest values of these parameters. On the other hand, the type and amount of added nuts had a statistically significant influence on the value of T_E_ (*p* < 0.001). The value of this parameter was the lowest in the control sample. The presence of nuts in the system in a statistically significant way increased the value of this parameter. The mean value of T_E_ for the WFH systems was higher than the control sample by only 0.1 °C, and in the case of WFW by as much as 1 °C. The retrograde enthalpy ΔH_R_ value for the control sample was the lowest and amounted to 0.5 J/g, and the addition of nuts to the system resulted in a significant increase in the value of this parameter. The two-way analysis of variance carried out confirmed only a statistically significant influence of the amount of nuts added on the value of this parameter (*p* < 0.001). The mean value of ΔH_R_ for WFH and WFW was almost three times higher than in the control sample. On the basis of ΔH_G_ and ΔH_R_, the degree of retrogradation (R%) was calculated, ranging from 8.5% (control) to 28.8% (WFW15%). According to many researchers [17,18,19] the thermal properties of starch are influenced by numerous factors, including the size and shape of starch grains, phosphorus content, length of amylopectin chains, presence, and content of non-starch components, and size of crystalline regions in starch grains.

### 3.2. Pasting Characteristics

**Figure 1:**
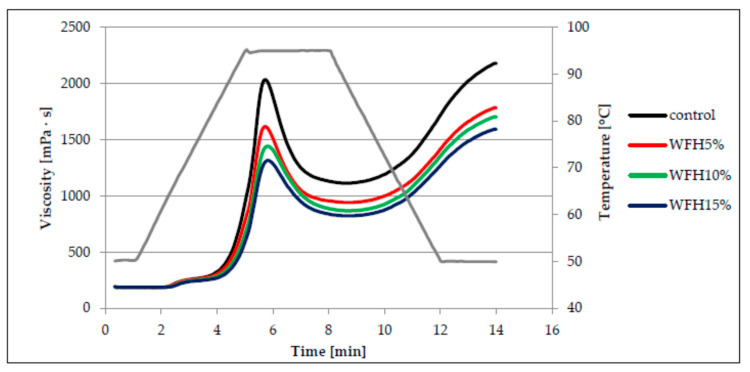
Pasting curves of wheat flour dispersion (control) and samples with hazelnuts addition.

**Figure 2:**
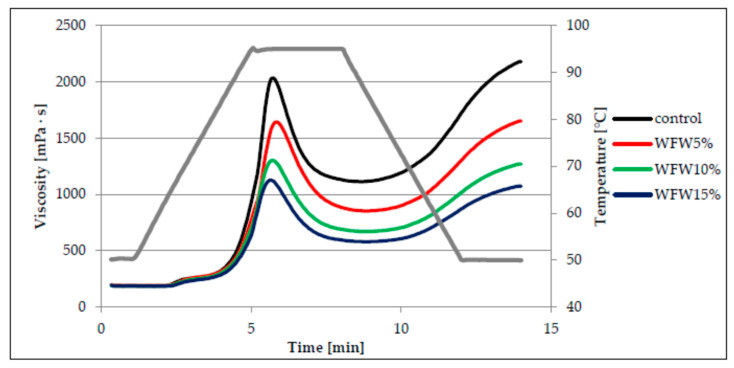
Pasting curves of wheat flour dispersion (control) and samples with walnuts additions.

The main component of flour responsible for the pasting process is starch. When starch is heated in hydrothermal conditions, the starch grains absorb water, swell, and then break, as a result of which amylose flows from their interior and the formation of a colloidal solution. The continuous phase of such a solution is water-soluble amylose. As a result of cooling such a system, an increase in viscosity is observed as a result of the formation of a three-dimensional network consisting of chains of amylose and amylopectin linked by hydrogen bonds, which is characterized by the ability to retain water [15,17,20]. Viscosity changes the analyzed systems of wheat flour with the addition of hazelnuts and walnuts in various proportions are illustrated in the form of the so-called pasting curves (Figure 1 and Figure 2). On the basis of their course, it can be stated that the addition of nuts had statistically significant influence on the pasting characteristics of the analyzed systems. The control sample, i.e., without the addition of nuts, had the highest peak, and thus the highest maximum viscosity. On the other hand, the presence of ground nuts in the system resulted in a significant reduction of the peak height and the reduction of the maximum viscosity value. The change was the greater with the greater the proportion of nuts was in the analyzed systems (Figure 1 and Figure 2). This may be due to the lower proportion of starch in systems with more ground nuts. Moreover, the course of the pasting curves indicates that replacing flour with walnuts (W) in the mixtures resulted in a greater reduction in maximum viscosity in comparison with hazelnuts (H). The viscosity peak is a measure of the free-swelling capacity of starch grains [20].

The parameters of the pasting characteristics are summarized in Table 2. Based on the analysis of research results, there was found a statistically significant influence of the amount of nuts added and the interaction between the type of nuts and the amount of their addition on the pasting temperature of the studied systems. The lowest value of the pasting temperature (PT) was found in the control sample (63.0 °C), while the systems with the addition of hazelnuts or walnuts pasted at a higher temperature, because the average PT value for the wheat flour system with the addition of H was 64.3 °C, and with the addition of W, it was 64.4 °C. Moreover, the value of PT increased, in a statistically significant way, with increasing proportion of nuts in the systems.

According to the researchers [21,22], the presence of fat and proteins, which bind amylose, hindering its transition into solution, increases the pasting temperature of starch. This is reflected in the systems studied in which the nuts present are a source of fat and protein. Moreover, according to Sandhu and Singh [23], high gelatinization temperature value is probably related to the considerable swelling resistance of starch grains. In the analyzed case, fat causes an isolating effect on the access of water. On the other hand, Song and Jane [24] indicate that phospholipids present in starch can form helical complexes with starch and thus reduce its water absorption, swelling capacity and, as a result, increase the gelatinization temperature of such a system. The control sample had the highest value of maximum viscosity (PV), while the type of nuts, the amount of their addition and the interactions between these factors had a statistically significant (*p* < 0.001) influence on the value of this parameter and the values of HPV, BD, FV, and SB parameters in the analyzed systems. The average PV value of the systems with the addition of hazelnuts and walnuts was lower by approximately 40% and 51%, respectively, in relation to the maximum viscosity of the control sample. The PV value decreased as the proportion of nuts in the system increased. The system with 15% share of W (1120 mPa·s) was characterized by the lowest maximum viscosity. This type of dependence results from the decreasing concentration of starch in the system, which is responsible for shaping the viscosity. In addition, the pasting process of amylose is hampered by the binding of fat to it. Nevertheless, the maximum viscosity of the starch paste is influenced by the ratio of amylose to amylopectin in starch, as starch with a higher amylopectin content is characterized by a higher maximum viscosity, which is related to its ability to swell in water while heating the paste [17,25,26]. In turn, Gupta et al. [27] and Zarzycki et al. [26] noted a positive linear correlation between the maximum viscosity of cereal starch paste and the content of amylose. As a result of further heating of the analyzed pastes, a significant decrease in viscosity occurred, the greater the greater the maximum viscosity they showed. The parameter of the pasting characteristics, which determines the decrease of viscosity during further heating of the paste, is BD, which had the highest values for the paste of the control sample, and the lowest for the paste based on WFH15%. Based on this parameter, it is also possible to evaluate the resistance of starch to high temperatures and shear forces [26]. It should be noted that the value of this parameter decreased with increasing share of nuts in the analyzed systems. A significant positive linear correlation was found between the BD parameter and the PV parameter (r = 0.99, *p* < 0.05). Cooling of the tested pastes resulted in a statistically significant increase in viscosity, with the final viscosities generally greater than the maximum viscosity. Only in the case of WFW10% and WFW15% systems, lower FV values were recorded compared to PV. The parameter determining the increase in viscosity during cooling is SB (*set back*). This parameter is also used to assess susceptibility of starch to retrogradation. Low SB value indicates poor susceptibility of starch to retrogradation [26]. The highest SB value was found in the paste based on the control sample (Table 2). In turn, the average value of the SB parameter for the systems with the addition of hazelnuts was 816 mPa·s, and for the systems with the addition of walnuts 628 mPa·s. The research showed a significant positive linear correlation between the values of the SB parameter and the values of the PV, HPV and FV parameters (r = 0.92, r = 0.99, r = 0.99, *p* < 0.05 respectively). According to Singh et al. [17], the starch gelatinization behavior in water systems depends on the physical and chemical properties of starch granules, such as the average grain size and shape, starch grain size distribution, amylose to amylopectin ratio or the content of non-starchy components, including minerals.

### 3.3. Viscoelastic Properties

**Figure 3:**
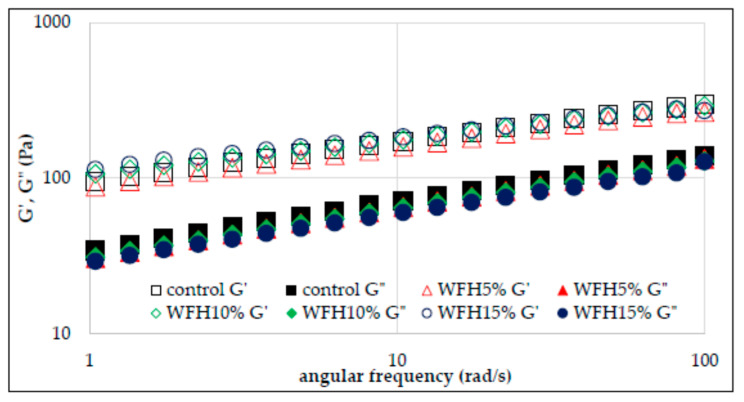
Sweep frequency curves of wheat flour gel and samples with hazelnuts addition.

**Figure 4:**
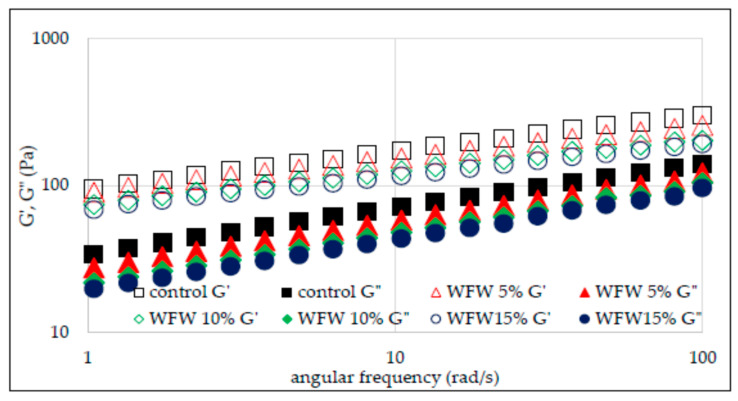
Sweep frequency curves of wheat flour gel and samples with walnuts addition.

**Figure 5:**
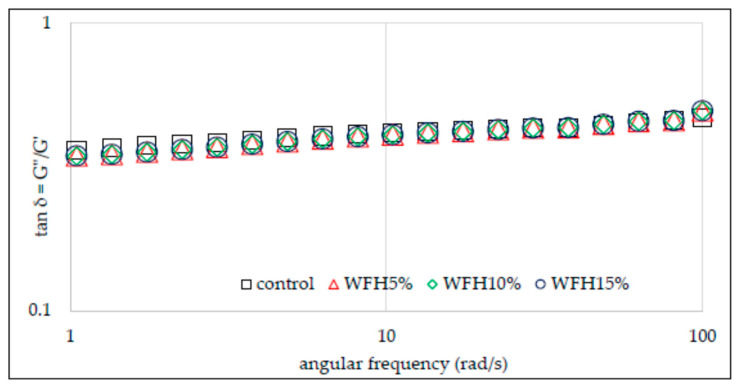
Tangent δ depending on the angular frequency of wheat flour gel and samples with hazelnuts addition.

**Figure 6:**
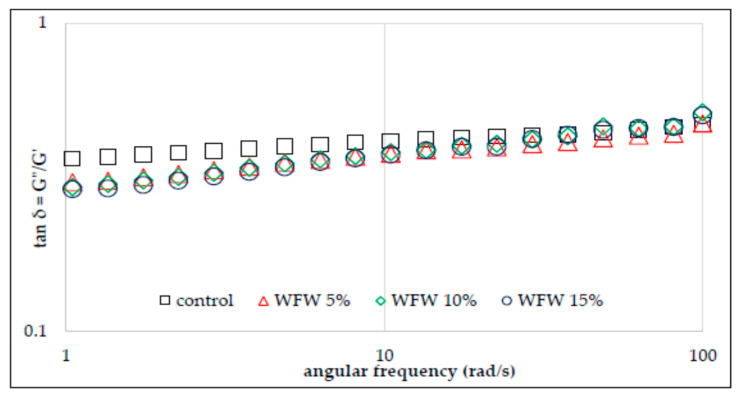
Tangent δ depending on the angular frequency of wheat flour gel and samples with walnuts addition.

The rheological properties of flour–nut systems were also analyzed by the small deformation rheology method with the use of an oscillating rheometer. The viscoelastic properties of the material result from the presence of certain structures capable of partially storing energy, which is partially recovered when the stress is released. However, at the same time, a large part of the same energy is irretrievably lost after the applied stress is released. Hence the conclusion that such materials are characterized by both elastic and viscous features. The use of an oscillating rheometer allows you to track changes in the value of conservative and loss moduli depending on temperature and frequency [19]. Thus, the conservation modulus (*G*′) describes the share of elastic properties in the analyzed material and corresponds to that part of the energy that is stored. In turn, the loss modulus (*G*″) characterizes part of the energy lost or scattered during sinusoidal deformation. The ratio of energy lost to stored, in each cycle, is described as tan δ, which otherwise informs about the physical behavior of the system [17,18,19]. Figure 3 and Figure 4 show the sweep frequency curves of systems with the addition of hazelnuts and walnuts, and Figure 5 and Figure 6 show changes in the tangent value of the phase shift angle with respect to the angular velocity for the analyzed systems. The values of the *G*′ and *G*″ modules depend on the amylose content in starch granules, the presence of non-starch components and the swelling capacity of the starch and its crystallinity.

It was found in the research that, in each of the analyzed cases, the values of the conservative modulus *G*′ were greater than the loss modulus *G*″, which indicates the domination of elastic properties over viscous ones. The presence of nuts in the analyzed systems and their growing share reduces the values of *G*′ and *G*″ moduli. However, the significant dependence of the values of moduli on the angular velocity and the tangent value of the phase shift angle (tan δ = *G*″/*G*′) indicate that the gels of the studied starches are weak gels, and the presence of nuts in the system deepened this tendency (Figure 5 and Figure 6). This is consistent with the observations of other authors [13]. The cited authors found a decrease in the value of *G*′ and *G*″ modules along with the increasing share of structured lipids in wheat dough. According to Watanabe et al. [28] and Agyare et al. [13], fat evenly distributes the gluten gel between the starch grains in the dough, thus reducing the friction between the starch granules and results in a reduction in the *G*′ and *G*″ values. Table 3 presents the values of the parameters of the power equations describing the mechanical spectra of the analyzed systems of wheat flour supplemented with hazelnuts and walnuts. The two-way analysis of variance showed a statistically significant influence of the type of nuts and of the interaction between the type of nuts and the amount of their addition to wheat flour (*p* < 0.001) on the value of the parameter *K*′ reflecting the initial value of *G*′ modulus.

The WFH15% system was characterized by the highest value of this parameter. In the case of systems with the addition of hazelnuts, the *K*′ value increased along with the increasing addition of H to the flour. The opposite situation was observed in the case of systems containing W, as in this case the value of *K*′ decreased with the increasing share of W in the system. Thus, the mean *K*′ value of WFW systems was 78.2 and was lower than the control sample and the mean of the WFH system by 24% and 32%, respectively. The two-way analysis of variance showed a statistically significant influence of the type of nuts, the amount of their addition and the interaction between the type of nuts and the amount of their addition to wheat flour (*p* < 0.001) on the value of the *K*″ parameter reflecting the initial value of *G*″ modulus. The value of the *K*″ parameter ranged from 18.9 (WFW15%) to 34.7 (control). The presence of nuts in the analyzed systems reduced the value of the described parameter. Moreover, in the case of both types of nuts, their increasing share in the system resulted in a decrease in the value of the *K*″ parameter. As a result of the statistical analysis, a significant negative linear correlation was found between the values of the *K*″ parameter and the temperature values characteristic for the pasting process T_O_ and T_E_ (r = −0.86, r = −0.87, *p* < 0.05 respectively), as well as a positive correlation with the parameters of the PV, HPV, FV, and SB pasting process (r = 0.81, r = 0.96, r = 0.97, r = 0.98, *p* < 0.05, respectively). A slight statistical differentiation among all analyzed gels was found for the values of the parameters *n*′ and *n*″ reflecting the sensitivity of the modules to changes in angular frequency.

### 3.4. Creep and Recovery Test

**Figure 7:**
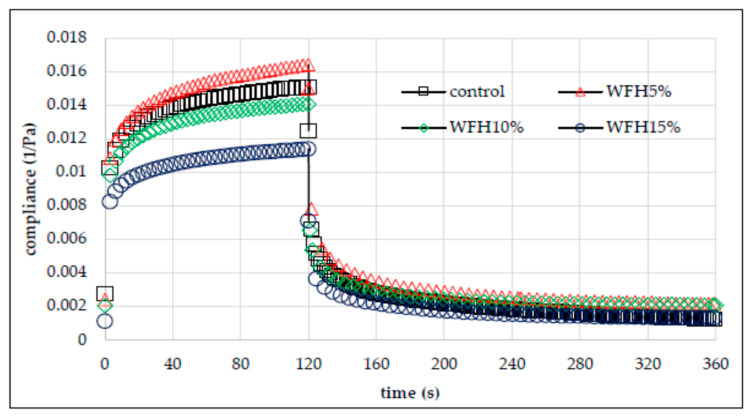
Creep and recovery curves of wheat flour gel and samples with hazelnuts addition.

**Figure 8:**
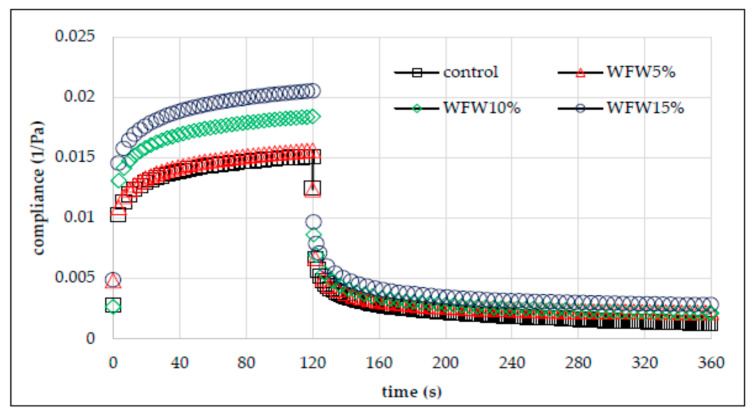
Creep and recovery curves of wheat flour gel and samples with walnuts addition.

The evaluation of the rheological properties of the analyzed wheat flour–nut systems also included a test describing the susceptibility of the system to deformation over time. The creep test measures the deformation of a material under applied stress. After it subsides, the degree of structural reconstruction is also measured using the recovery test [29,30]. Creep and recovery phenomenon is related to the temporary reorientation of chemical bonds that build the viscoelastic structure in the material. The temporary susceptibility of the bond orientation in an elastic material is related to the applied stress, which, however, disappears as soon as it wears off. In turn, the characteristics of the viscoelasticity of the material result from the disturbance and conversion of chemical bonds [30,31]. Figure 7 and Figure 8 show exemplary creep and recovery plots of the analyzed systems.

All the curves have shape characteristic for viscoelastic materials. Based on their course, it can be concluded that the WFW15% gel was the most susceptible to deformation. In turn, the gel based on WFH15% was the most resistant to deformation over time. Thus, the nature of the effect of adding nuts to wheat flour is not clear. In general, the addition of hazelnuts increased the deformation resistance of the system, while the addition of walnuts had a different effect. Table 4 summarizes the parameters of the Burger’s model used to describe the susceptibility to deformation of the analyzed systems. The two-way analysis of variance showed a statistically significant influence of the type of nuts and the interaction between the type of nuts and the amount of their addition to wheat flour (*p* < 0.001) on the value of the immediate and viscoelastic susceptibility parameters. Immediate compliance (*J*_0_) is related to the tensile energy of elastic bonds, which disappears as soon as the force is released. The value of this parameter ranged from 0.0059 Pa^−1^ (WFH15%) to 0.0105 Pa^−1^ (WFW15%). The mean value of the *J*_0_ parameter for the WFH and WFW systems was respectively lower and higher by 17% and 12% in relation to the control sample. Thus, the addition of hazelnuts reduced the deformability, and the addition of walnuts increased it. In turn, the viscoelasticity (*J*_1_) is related to the destruction or transformation of bonds in the material. The value of this parameter ranged from 0.0041 Pa^−1^ (WFH15%) to 0.0082 Pa^−1^ (WFW10%). The value of the parameter η_0_ was significantly influenced by the type of nuts, the amount of their addition, and the interactions between these factors (*p* < 0.001). It was found that the systems with the addition of nuts were characterized by a lower value of this parameter in comparison with the control sample. The mean value of *η*_0_ of the WFH and WFW systems was lower by 15% and 34%, respectively, in relation to the control sample. The change in the deformation resistance of the sample over time is probably due to changes in the water absorption of the flour/system due to the addition of nuts that contain fat, which is a hydrophobic substance. According to Witczak et al. [31], the increase in the resistance of the dough to deformation over time may be due to the increased swelling capacity of starch. Moreover, according to Hüttner et al. [32], the flexibility of the dough is related to its hydration capacity, resulting from the size of flour particles, the degree of starch damage, protein content, and the presence of non-starchy and hydrophobic substances. The retardation time characterizes the time required for the viscoelastic material to respond to an applied stress [31]. The highest value of this parameter was characteristic for the control sample (13.83 s), and the lowest for the WFW10% system. As a result of the statistical analysis, significant linear correlations were found between the *J*_0_ and *J*_1_ parameters with the *K*′ parameters (r = −0.84, r = −0.91, *p* < 0.05 respectively) and *n*″ (r = 0.79, r = 0.78, *p* < 0.05, respectively). Moreover, the retardation time *λ_ret_* negatively correlated with the values of the retrogradation degree R (r = −0.77, *p* < 0.05).

## 4. Conclusions

The results of the research indicate that the addition of hazelnuts and walnuts influenced the thermal and rheological properties of these systems. It was shown that the systems containing nuts were characterized by a higher gelatinization temperature and had a higher gelatinization and retrogradation enthalpy. Moreover, the degree of retrogradation was about three times higher in systems where some of the flour was replaced with H and W. In turn, the presence of nuts in the experimental systems significantly decreased their maximum viscosity. The decrease was the greater the greater the share of nuts. The system with 15% walnuts was characterized by the lowest viscosity. Similarly, the systems containing nuts were characterized by lower values of the BD, FV, and SB parameters in comparison with the control sample. The share of nuts in the analyzed systems influenced their viscoelastic properties because the systems with the addition of H and W had lower *G*′ and *G*″ values, and the gels obtained on their basis were weak gels. Moreover, based on the results of the creep and recovery test, it was shown that the systems containing walnuts were more susceptible to deformation over time than the systems with the addition of hazelnuts. The analysis of the research results shows that replacing flour with hazelnuts or walnuts significantly affects the thermal and rheological properties of such systems, changing the water absorption of the flour. The optimal addition of nuts should take into account the changes in water absorption of the flour.

## Figures and Tables

**Table 1:** Thermodynamic characteristics of gelatinization and retrogradation of systems wheat flour with nuts.

Sample	Gelatinization					Retrogradation					
	T_O_ (°C)	T_P_ (°C)	T_E_ (°C)	ΔT (°C)	ΔH_G_ (J/g)	T_O_ (°C)	T_P_ (°C)	T_E_ (°C)	ΔT (°C)	ΔH_R_ (J/g)	R (%)
control	59.3 ^a^ ± 0.4	64.8 ^a^ ± 0.4	69.7 ^a^ ± 0.4	10.4 ^cd^ ± 0.6	6.1 ^a^ ± 0.1	45.4 ^a^ ± 0.2	50.2 ^b^ ± 1.1	52.7 ^ab^ ± 0.9	7.4 ^a^ ± 1.1	0.5 ^a^ ± 0.0	8.5 ^a^
WFH5%	60.0 ^b^ ± 0.2	65.0 ^b^ ± 0.4	70.5 ^b^ ± 0.3	10.5 ^cd^ ± 0.2	6.8 ^cd^ ± 0.2	44.8 ^b^ ± 0.1	49.0 ^a^ ± 0.6	52.3 ^a^ ± 0.3	7.5 ^a^ ± 0.2	1.6 ^b^ ± 0.0	23.3 ^b^
WFH10%	60.3 ^b^ ± 0.0	65.2 ^bc^ ± 0.3	70.7 ^bc^ ± 0.3	10.4 ^cd^ ± 0.3	7.0 ^de^ ± 0.0	45.1 ^b^ ± 0.1	49.2 ^a^ ± 0.6	52.8 ^ab^ ± 0.5	7.7 ^ab^ ± 0.4	1.8 ^c^ ± 0.0	25.2 ^bc^
WFH15%	60.3 ^b^ ± 0.2	65.4 ^bcd^ ± 0.2	71.2 ^c^ ± 0.2	10.9 ^d^ ± 0.3	7.2 ^e^ ± 0.1	44.9 ^b^ ± 0.1	49.6 ^a^ ± 0.2	53.4 ^bc^ ± 0.5	8.5 ^cd^ ± 0.5	2.0 ^d^ ± 0.0	28.1 ^d^
WFW5%	61.2 ^c^ ± 0.4	65.8 ^d^ ± 0.4	70.7 ^bc^ ± 0.6	9.5 ^a^ ± 0.4	6.3 ^b^ ± 0.0	45.0 ^b^ ± 0.1	49.7 ^a^ ± 0.3	53.3 ^bc^ ± 0.0	8.3 ^bc^ ± 0.1	1.6 ^b^ ± 0.0	25.3 ^bc^
WFW10%	61.0 ^c^ ± 0.1	65.7 ^cd^ ± 0.1	70.9 ^bc^ ± 0.1	9.9 ^ab^ ± 0.2	6.6 ^bc^ ± 0.1	44.9 ^b^ ± 0.3	49.8 ^a^ ± 0.2	53.9 ^c^ ± 0.7	9.0 ^cd^ ± 0.6	1.8 ^c^ ± 0.0	27.3 ^cd^
WFW15%	61.2 ^c^ ± 0.1	65.8 ^d^ ± 0.2	71.3 ^c^ ± 0.1	10.1 ^bc^ ± 0.1	6.9 ^de^ ± 0.3	44.8 ^b^ ± 0.3	49.4 ^a^ ± 0.5	53.9 ^c^ ± 0.8	9.1^d^ ± 0.5	1.9 ^d^ ± 0.1	28.8 ^d^
	**two-factor ANOVA–p**										
Factor 1	<0.001	<0.001	0.326	<0.001	<0.001	0.493	0.064	<0.001	<0.001	0.758	<0.001
Factor 2	0.228	0.426	<0.001	<0.001	<0.001	0.383	0.764	<0.001	<0.001	<0.001	<0.001
Factor 1 × factor 2	0.080	0.526	0.963	0.232	0.355	0.140	0.201	0.581	0.309	0.747	0.605

Mean values from three repetitions ± SD; T_O_—onset temperature, T_P_—peak temperature, T_E_—endset temperature, ΔH_G_—enthalpy of gelatinization, ΔH_R_—enthalpy of retrogradation, R—percentage of retrogradation = (ΔH_R_/ΔH_G_) × 100. Values in columns followed by the same superscript letters do not significantly differ at significance level of 0.05. Factor 1—type of nuts. Factor 2—the supplementation level. Factor 1 × factor 2—interactions between type of nuts and the supplementation level.

**Table 2:** Pasting characteristics of systems wheat flour with nuts.

Sample	PT (°C)	PV (mPa·s)	HPV (mPa·s)	BD (mPa·s)	FV (mPa·s)	SB (mPa·s)
control	63.0 ^a^ ± 0.2	2039 ^e^ ± 3	1113 ^f^ ± 8	926 ^f^ ± 4	2178 ^g^ ± 8	1065 ^f^ ± 1
WFH5%	63.3 ^b^ ± 0.3	1619 ^d^ ± 3	941 ^e^ ± 8	678 ^d^ ± 6	1784 ^f^ ± 16	843 ^e^ ± 8
WFH10%	64.6 ^cd^ ± 0.4	1442 ^c^ ± 7	868 ^d^ ± 10	574 ^b^ ± 10	1703 ^e^ ± 13	834 ^e^ ± 3
WFH15%	65.0 ^d^ ± 0.3	1315 ^b^ ± 35	822 ^c^ ± 15	493 ^a^ ± 20	1593 ^c^ ± 36	771 ^c^ ± 21
WFW5%	64.6 ^cd^ ± 0.4	1642 ^d^ ± 32	853 ^d^ ± 13	789 ^e^ ± 19	1651 ^d^ ± 29	798 ^d^ ± 17
WFW10%	64.0 ^cd^ ± 0.0	1302 ^b^ ± 12	670 ^b^ ± 3	632 ^c^ ± 10	1270 ^b^ ± 6	600 ^b^ ± 4
WFW15%	64.8 ^d^ ± 0.0	1120 ^a^ ± 18	576 ^a^ ± 6	544 ^b^ ± 12	1062 ^a^ ± 13	486 ^a^ ± 7
	**two-factor ANOVA–p**					
Factor 1	0.459	<0.001	<0.001	<0.001	<0.001	<0.001
Factor 2	<0.001	<0.001	<0.001	<0.001	<0.001	<0.001
Factor 1 × factor 2	<0.001	<0.001	<0.001	<0.001	<0.001	<0.001

Mean values from three repetitions ± SD; PT—pasting temperature, PV—peak viscosity, HPV—hot paste viscosity, BD—breakdown (PV—HPV), FV—final viscosity, SB—setback (FV—HPV). Values in columns followed by the same superscript letters do not significantly differ at significance level of 0.05. Factor 1—type of nuts. Factor 2—the supplementation level. Factor 1 × factor 2—interactions between type of nuts and the supplementation level.

**Table 3:** Parameters of power law equations describing viscoelastic properties (25 °C) of systems wheat flour with nuts.

Sample	*K*′	*n*′	R^2^	*K*″	*n*″	R^2^
control	97.0 ^c^ ± 5.0	0.25 ^c^ ± 0.02	0.9989	34.7 ^f^ ± 1.0	0.30 ^a^ ± 0.01	0.9993
WFH5%	89.1 ^b^ ± 0.8	0.25 ^c^ ± 0.00	0.9981	30.7 ^e^ ± 0.2	0.32 ^a^ ± 0.00	0.9985
WFH10%	106.2 ^d^ ± 3.5	0.22 ^b^ ± 0.01	0.9992	31.1 ^e^ ± 0.6	0.31 ^a^ ± 0.00	0.9991
WFH15%	115.9 ^e^ ± 4.9	0.20 ^a^ ± 0.00	0.9949	29.1 ^d^ ± 1.0	0.31 ^a^ ± 0.01	0.9969
WFW5%	90.6 ^b^ ± 1.2	0.23 ^b^ ± 0.01	0.9995	27.5 ^c^ ± 0.3	0.32 ^a^ ± 0.00	0.9993
WFW10%	74.7 ^a^ ± 1.7	0.22 ^b^ ± 0.00	0.9989	21.8 ^b^ ± 0.5	0.33 ^b^ ± 0.00	0.9975
WFW15%	69.4 ^a^ ± 2.0	0.22 ^b^ ± 0.00	0.9991	18.9 ^a^ ± 0.8	0.34 ^b^ ± 0.00	0.9991
	**two-factor ANOVA–p**					
Factor 1	<0.001	0.282		<0.001	<0.001	
Factor 2	0.305	<0.001		<0.001	0.080	
Factor 1 × factor 2	<0.001	<0.001		<0.001	<0.001	

Mean values from three repetitions ± SD; *K*—consistency coefficient, *n*—flow behavior index, *K*′, *K*″, *n*′, *n*″—power low equations constants. Values in columns followed by the same superscript letters do not significantly differ at significance level of 0.05. Factor 1—type of nuts. Factor 2—the supplementation level. Factor 1 × factor 2—interactions between type of nuts and the supplementation level.

**Table 4:** Values of the Burgers model parameters for creep and recovery curves of the systems wheat flour with nuts.

Sample	*J*_0_ (×10^−3^) (Pa^−1^)	*J*_1_ (×10^−3^) (Pa^−1^)	*η*_0_ (×10^4^) (Pa·s)	*λ_ret_* (s)	R^2^
control	7.65 ^c^ ± 0.41	5.99 ^bc^ ± 0.40	6.71 ^d^ ± 0.26	13.83 ^c^ ± 1.48	0.9874
WFH5%	6.90 ^b^ ± 0.50	7.28 ^cd^ ± 0.42	4.63 ^b^ ± 0.12	10.26 ^b^ ± 1.05	0.9874
WFH10%	6.67 ^b^ ± 0.37	5.44 ^ab^ ± 0.36	5.18 ^c^ ± 0.10	8.06 ^ab^ ± 0.84	0.9870
WFH15%	5.92 ^a^ ± 0.32	4.14 ^a^ ± 0.00	7.66 ± 0.23	10.66 ^b^ ± 1.27	0.9887
WFW5%	7.62 ^c^ ± 0.36	5.92 ^bc^ ± 0.36	4.84 ^bc^ ± 0.71	9.31 ^ab^ ± 0.89	0.9923
WFW10%	7.91 ^c^ ± 0.58	8.21 ^e^ ± 0.57	4.60 ^b^ ± 0.13	6.71 ^a^ ± 0.29	0.9892
WFW15%	10.56 ^d^ ± 0.48	7.30 ^cd^ ± 0.00	3.73 ^a^ ± 0.78	9.76 ^ab^ ± 0.98	0.9982
	**two-factor ANOVA–p**				
Factor 1	<0.001	<0.001	<0.001	0.213	
Factor 2	0.114	0.082	<0.001	<0.001	
Factor 1 × factor 2	<0.001	<0.001	<0.001	0.967	

Mean values from three repetitions ± SD. Values in columns followed by the same superscript letters do not significantly differ at significance level of 0.05. Factor 1—type of nuts. Factor 2—the supplementation level. Factor 1 × factor 2—interactions between type of nuts and the supplementation level.

## Data Availability

All data is included in the article.

## Sample Availability

Not available.
